# Use of individualized starting dose and niraparib hematologic adverse event management costs in ovarian cancer

**DOI:** 10.57264/cer-2024-0133

**Published:** 2024-12-06

**Authors:** Whitney S Graybill, Ignace Vergote, Bhavana Pothuri, Maarit Anttila, David M O'Malley, Domenica Lorusso, Ashley F Haggerty, Michel Fabbro, John K Chan, Florian Heitz, Lyndsay J Willmott, Ilan Bruchim, Ying Zhuo, Purificación Estévez-García, Bradley J Monk, Hannelore Denys, Anja Knudsen, Anna V Tinker, Luis Manso Sánchez, Diane Provencher, Maria Pilar Barretina-Ginesta, John Hartman, Donna V Booth, Antonio González-Martín

**Affiliations:** 1 Division of Gynecologic Oncology, Medical University of South Carolina, Charleston, SC 29425, USA; 2 University Hospitals Leuven, Leuven Cancer Institute, and Belgium & Luxembourg Gynaecological Oncology Group (BGOG), Leuven, B912 3000, Belgium; 3 GOG Foundation and Laura & Isaac Perlmutter Cancer Center, NYU Langone Health, New York, NY 10016, USA; 4 Kuopio University Hospital, Kuopio, Finland, and NSGO, Copenhagen, 9 70200, Denmark; 5 The Ohio State University andthe James Comprehensive Cancer Center, Columbus, OH 43210, USA; 6 Humanitas San Pio X, Milan, Humanitas University, Rozzano, 20089, Italy; 7 Hackensack Meridian Health, Hackensack, NJ 07601, USA; 8 Institut Régional du Cancer de Montpellier, Montpellier, and GINECO, Paris, 34298, France; 9 California Pacific Medical Center Palo Alto Medical Foundation, Sutter Cancer Research Consortium, San Francisco, CA 94301, USA; 10 AGO Study Group and Kliniken Essen-Mitte, Essen, and Charité - Universitätsmedizin Berlin, Humboldt-Universität zu Berlin, Berlin Institute of Health, Department of Gynaecology, Berlin, 12203, Germany; 11 Arizona Center for Cancer Care, Phoenix, AZ 85027, USA; 12 Hillel Yaffe Medical Center, Hadera, Technion Institute of Technology, Haifa, 38100, Israel, & ISGO; 13 Kadlec Hematology and Oncology Clinic, Richland, WA 99336, USA; 14 Hospital Universitario Virgen del Rocío, Sevilla, and GEICO, Madrid, 41013, Spain; 15 GOG Foundation; Florida Cancer Specialists and Research Institute, West Palm Beach, FL 33401, USA; 16 Ghent University Hospital, Ghent, 9000, Belgium; 17 Odense University Hospital, Odense, 5000, Denmark; 18 BC Cancer Vancouver, and Department of Medicine, University of British Columbia, Vancouver, BC, V6T1Z1, Canada; 19 Hospital Universitario 12 de Octubre & GEICO, Madrid, 28041, Spain; 20 Centre Hospitalier de l'Université de Montréal, Université de Montréal and Institut du Cancer de Montréal, Montreal, QC, H3T1J4, Canada; 21 Medical Oncology Department, Institut Català d'Oncologia; Girona Biomedical Research Institute (IDIBGI-CERCA), Girona University, Girona, Spain, and GEICO, 08908, Spain; 22 GSK, Philadelphia, PA 19112, USA; 23 GSK, Durham, NC 27709, USA; 24 Medical Oncology Department, Program in Solid Tumours, CIMA, Cancer Center Clínica Universidad de Navarra, Madrid, and Grupo Español de Investigación en Cancer ginecológicO (GEICO), Madrid, 28027, Spain

**Keywords:** cost management, maintenance therapy, niraparib, ovarian cancer, PARP inhibitor

## Abstract

**Aim:**

To understand the impact of the niraparib individualized starting dose (ISD), compared with fixed starting dose (FSD), on the cost of hematologic adverse event (AE) management from a US payer perspective.

**Methods:**

The frequencies of grade ≥3 hematologic AEs that occurred in >1% of patients treated with niraparib were obtained from the primary analysis results of the phase III PRIMA/ENGOT-OV26/GOG-3012 trial. US unit costs for each grade ≥3 AE in the base case were obtained from the 2017 Agency for Healthcare Research and Quality Healthcare Cost and Utilization Project database; unit costs were adjusted to 2020 US dollars. AE management costs per patient were calculated by multiplying AE unit cost by the frequency of each AE by niraparib starting dose. Because AEs were assumed to occur independently of one another, costs were added to derive the total cost.

**Results:**

For niraparib, the estimated AE management cost per patient was lower for the ISD than the FSD for all hematologic AEs (FSD vs ISD: thrombocytopenia, $4701.87 vs $1921.89; anemia, $2784.00 vs $1760.59; platelet count decreased, $2103.47 vs $922.51; neutropenia, $2112.50 vs $1369.56; neutrophil count decreased, $1285.87 vs $770.38). The total mean calculated AE management cost per patient was $12,987.71 with the FSD and $6744.93 with the ISD.

**Conclusion:**

For niraparib, the cost of managing hematologic AEs in the US was reduced by almost half with the ISD compared with the FSD. The cost reduction and improvements in safety associated with the niraparib ISD support its use in clinical practice.

## Plain language summary

How using an individualized dose of niraparib influences the cost of managing blood cell-related side effects.

**What is this article about?:**

Patients with newly diagnosed advanced ovarian cancer that responds to platinum-based chemotherapy may receive niraparib maintenance therapy to extend the time before the cancer comes back or gets worse. The niraparib starting dose can be fixed (same amount for everyone) or individualized based on body weight and platelet count.

**What were the results?:**

In this US-based analysis, using the niraparib individualized starting dose (ISD) reduced the cost of managing blood cell-related side effects compared with using the fixed starting dose (FSD).

**What do the results mean?:**

Using the niraparib ISD reduced the cost of managing blood cell-related side effects compared with the FSD. Results of other past studies have also shown that using the niraparib ISD can reduce side effects and can treat the cancer just as effectively, when compared with the FSD. Along with the results of those past studies, the cost reductions observed in this study support using the niraparib ISD as maintenance therapy in patients with newly diagnosed advanced ovarian cancer that responds to platinum-based chemotherapy.

## Shareable abstract

Researchers of the US-based analysis found that niraparib dosing based on patient body weight and platelet count was associated with nearly 50% lower costs for managing hematologic adverse events, compared with the fixed starting dose, for first-line maintenance treatment of patients with advanced epithelial ovarian cancer. These findings highlight the cost reduction and safety improvements associated with individualized starting doses of niraparib, further supporting its use in clinical practice.

Worldwide, ovarian cancer is the eighth leading cause of cancer-related deaths among women [1]. In the US, estimates indicate that there will be more than 19,600 new cases of ovarian cancer diagnosed in 2024 alone [2]. In addition to initial treatment with surgery and platinum-based chemotherapy, the treatment landscape for patients with advanced ovarian cancer has expanded to include maintenance treatment with poly(ADP)-ribose polymerase (PARP) inhibitors, alone or in combination with bevacizumab [3,4]. Maintenance treatment with the PARP inhibitor niraparib has been shown to improve progression-free survival in patients with newly diagnosed advanced epithelial ovarian cancer that responded to first-line platinum-based chemotherapy [5–7].

Consistent with other PARP inhibitors, niraparib is known to cause hematologic adverse events (AEs), particularly early during treatment [8–10]. In the ENGOT-OV16/NOVA trial (NCT01847274) of niraparib maintenance therapy in patients with platinum-sensitive recurrent epithelial ovarian cancer, all patients received a fixed starting dose (FSD) of 300 mg once daily [11]. The niraparib FSD was associated with grade ≥3 treatment-emergent AEs (TEAEs) of thrombocytopenia, anemia and neutropenia, with a high incidence of TEAEs resulting in dose reduction [11]. Results from subsequent analyses of the NOVA trial revealed that low baseline body weight (<77 kg) and platelet count (<150,000/μl) were associated with an increased incidence of grade ≥3 thrombocytopenia events, indicating that these patients could benefit from a lower starting dose of niraparib [12].

To improve patient safety and reduce the incidence of hematologic AEs, the starting dose of niraparib was adjusted in a portion of the patients included in the PRIMA/ENGOT-OV26/GOG-3012 trial (NCT02655016) of niraparib first-line maintenance [5]. The PRIMA protocol was amended partway through enrollment to introduce an individualized starting dose (ISD), in which patients with baseline body weight <77 kg or baseline platelet count <150,000/μl received 200 mg once daily and patients with baseline body weight ≥77 kg and baseline platelet count ≥150,000/μl received 300 mg once daily [5]. Results demonstrated that introduction of the ISD decreased the incidence of hematologic TEAEs while maintaining the progression-free survival benefit of niraparib compared with placebo [5,13]. Based on the results from the PRIMA trial, the niraparib ISD was approved for first-line maintenance therapy in the US [14]. However, the economic impact of the introduction of the niraparib ISD on the cost of hematologic AE management remains unexplored.

## Methods

### Evaluation of the niraparib ISD in the PRIMA/ENGOT-OV26/GOG-3012 trial

Niraparib first-line maintenance treatment was evaluated in the phase III randomized double-blind, placebo-controlled PRIMA trial in patients with newly diagnosed advanced epithelial ovarian cancer that responded to first-line platinum-based chemotherapy [5]. Detailed information on the study design and eligibility criteria have been described previously [5]. In the PRIMA trial, 2 different niraparib starting doses were used, the FSD and the ISD. At study start (July 2016), all patients received an FSD of 300 mg once daily. In November 2017, the protocol was amended so that newly enrolled patients received an ISD based on baseline body weight and platelet count (200 mg once daily in patients with baseline body weight <77 kg or baseline platelet count <150,000/μl and 300 mg once daily in all other patients) [5]. The primary analysis results from the PRIMA trial were published in 2019 and included an assessment of safety by niraparib starting dose [5]; these results are used for this analysis (clinical cut-off date, 17 May 2019).

### Selection of hematologic AEs for assessment

This cost analysis included grade ≥3 hematologic AEs that occurred in >1% of patients in the niraparib arm in the PRIMA primary analysis results: thrombocytopenia, anemia, platelet count decreased, neutropenia and neutrophil count decreased [5]. Grade ≥3 events were selected for analysis because they were the most likely to require hospitalization and interventions such as transfusions for management because of their severity.

### Frequencies of AEs

If a patient experienced ≥1 event within a given preferred term, that patient was counted only once for that term. Unrounded frequencies of grade ≥3 AEs for patients treated with the niraparib ISD and FSD were used for calculations.

### Cost calculations

The unit costs in the US for the hospital-related management of each grade ≥3 AE in the base case were obtained from the 2017 Agency for Healthcare Research and Quality Healthcare Cost and Utilization Project (HCUP) National (Nationwide) Inpatient Sample (NIS) database [15]. The all-payer HCUP NIS database is nationally representative and contains data from more than 7 million community hospital stays each year; the NIS database does not include rehabilitation and long-term acute care hospitals [16,17]. Unit costs were adjusted to 2020 US dollars, and AE management costs per patient were calculated by multiplying AE unit cost by the unrounded frequency of each AE by niraparib starting dose. Because AEs were assumed to occur independently of one another, their costs were added to derive the total cost.

## Results

**Figure 1.**
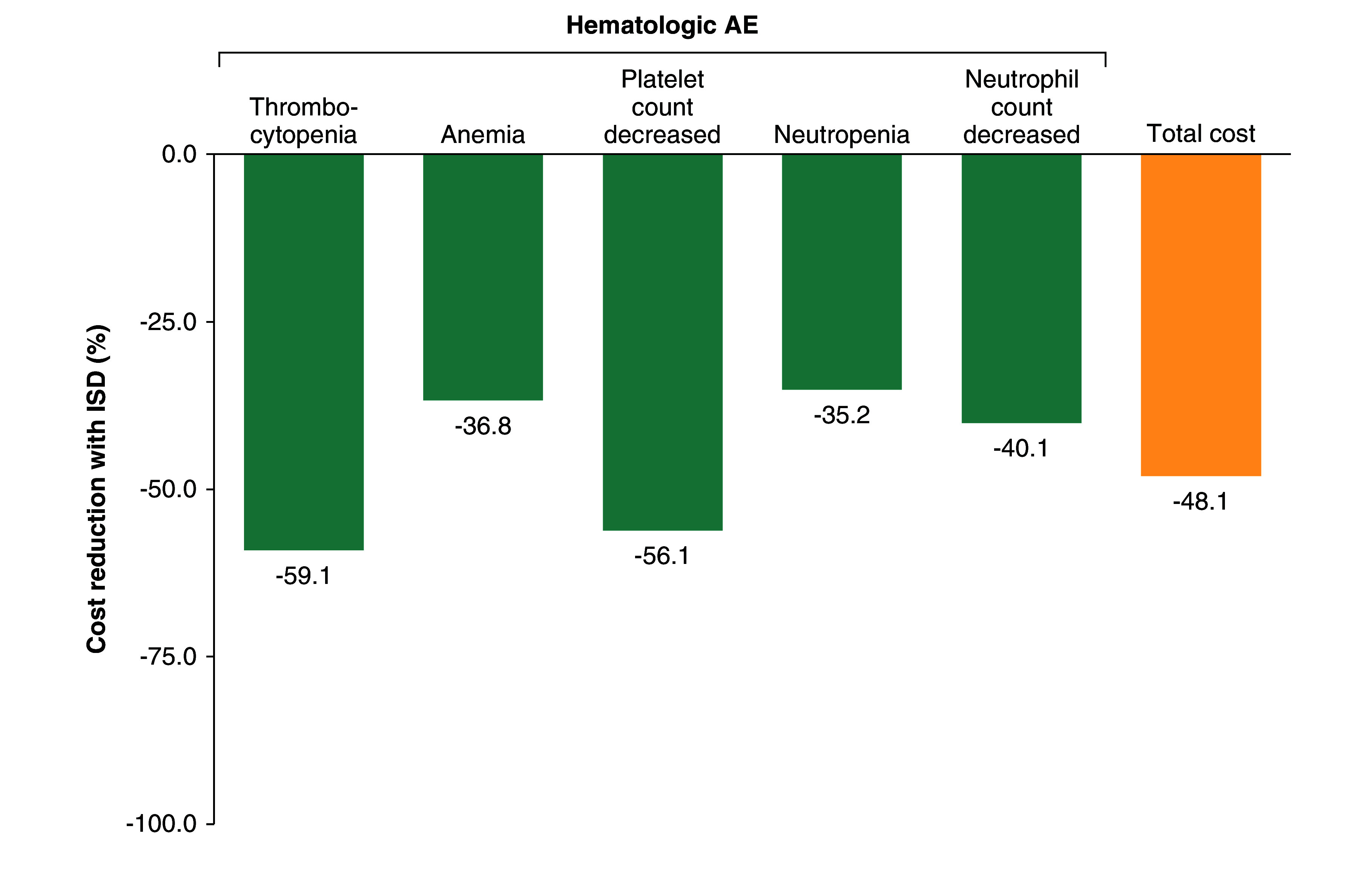
Hematologic AE management cost reduction with the niraparib individualized starting dose. Percent reduction in the cost of hematologic AE management with the niraparib ISD. AE: Adverse event; ISD: Individualized starting dose.

For niraparib, the estimated AE management cost per patient in the US was lower for the ISD than for the FSD for all hematologic AEs (Tables 1 & 2). The total mean calculated cost per patient was $12,987.71 with the FSD and $6744.93 with the ISD (Table 1). The percent reduction in the cost of hematologic AE management with the niraparib ISD compared with the FSD was also calculated (Figure 1). Thrombocytopenia had the greatest cost reduction (59.1%) of evaluated hematologic AEs. Implementing the niraparib ISD reduced the total cost of hematologic AE management by 48.1% as compared with the FSD.

**Table 1.** Estimated cost of hematologic AE management using the May 2019 data cut-off date.

Hematologic AE	Frequency of grade ≥3 AE in patients treated with niraparib, n (%)		AE unit cost^†^	Mean calculated cost per patient^‡^	
	FSD (n = 315)	ISD (n = 169)		FSD	ISD
Thrombocytopenia	114 (36.2)	25 (14.8)	$12,992.00	$4701.87	$1921.89
Anemia	112 (35.6)	38 (22.5)	$7830.00	$2784.00	$1760.59
Platelet count decreased	51 (16.2)	12 (7.1)	$12,992.00	$2103.47	$922.51
Neutropenia	46 (14.6)	16 (9.5)	$14,466.00	$2112.50	$1369.56
Neutrophil count decreased	28 (8.9)	9 (5.3)	$14,466.00	$1285.87	$770.38
Total cost				$12,987.71	$6744.93

† Cost in the US adjusted to 2020 US dollars.

‡ Costs calculated using the unrounded frequency of grade ≥3 AEs.

AE: Adverse event; FSD: Fixed starting dose; ISD: Individualized starting dose.

**Table 2.** Estimated cost of hematologic AE management using November 2021 data cut-off date.

Hematologic AE	Frequency of grade ≥3 AE in patients treated with niraparib, n (%)		AE unit cost^†^	Mean calculated cost per patient^‡^	
	FSD (n = 315)	ISD (n = 169)		FSD	ISD
Thrombocytopenia^§^	155 (49.2)	37 (21.9)	$12,992.00	$6392.82	$2844.40
Anemia	114 (36.2)	39 (23.1)	$7830.00	$2833.71	$1806.92
Neutropenia^¶^	78 (24.8)	25 (14.8)	$14,466.00	$3582.06	$2139.94
Total cost				$12,808.66	$6791.27

† Cost in the US adjusted to 2020 US dollars.

‡ Costs calculated using the unrounded frequency of grade ≥3 AEs.

§ Includes platelet count decreased.

¶ Includes neutrophil count decreased.

AE: Adverse event; FSD: Fixed starting dose; ISD: Individualized starting dose.

Confirmatory calculations were performed using grouped terms for thrombocytopenia (thrombocytopenia and platelet count decreased), anemia (anemia, hemoglobin decreased, red blood cell decreased, hematocrit decreased and anemia macrocytic) and neutropenia (neutropenia, neutrophil count decreased, febrile neutropenia and neutropenic sepsis) using published findings from 17 November 2021, clinical cut-off date, with 3.5 years of follow-up [6]. Results were similar, with AE management cost reductions of 55.5%, 36.2% and 40.3% observed with the ISD compared with the FSD for thrombocytopenia, anemia and neutropenia, respectively (Table 2).

## Discussion

In patients with advanced ovarian cancer, PARP inhibitor maintenance therapy represents an important treatment option after a response to first-line treatment [3,4]. The safety profiles of the 3 PARP inhibitors approved for use in the US vary, but as a class, PARP inhibitors have been associated with the hematologic AE of anemia [18]. Niraparib is also associated with increased incidences of thrombocytopenia and neutropenia [10,18]. In the PRIMA trial, introduction of the niraparib ISD reduced the incidence of any-grade and grade ≥3 events of thrombocytopenia, anemia and neutropenia [5,13]. A detailed assessment of the safety and tolerability of the niraparib ISD in the PRIMA trial found that first events of thrombocytopenia, anemia and neutropenia occurred early during treatment, had a short duration (≈2 weeks) and resolved in ≥90% of patients [19]. On the basis of the ISD findings in the PRIMA trial, the ISD is the current globally approved dosing for niraparib first-line maintenance [14,20,21].

In this analysis, the use of the niraparib ISD reduced the cost of managing severe hematologic AEs compared with the FSD. Reductions were observed for all hematologic AEs examined, and the total cost reduction as compared with the FSD was more than 48%. The reduced cost associated with the ISD is notable and supports the use of the ISD from a US payer perspective.

## Limitations

Several limitations must be considered when interpreting these findings. First, the PRIMA trial was a global, phase III clinical trial with specific eligibility criteria. Accordingly, the niraparib FSD and ISD results for hematologic AEs may not be representative of the experience of patients with advanced ovarian cancer in the USA treated in everyday clinical practice. The small sample size (n = 169) for the niraparib ISD should also be considered. Additionally, the HCUP database is designed to capture costs for hospital-related care and does not capture costs for care provided in physician offices or outside laboratories. Similar to other large, nationwide databases, the HCUP database is also subject to data entry errors and incomplete reporting. Last, it is important to note that these are estimated costs, and the actual costs of managing grade ≥3 hematologic AEs may vary depending on circumstances.

## Conclusion

For niraparib, the cost of managing severe (ie, grade ≥3) hematologic AEs in the US was reduced for the ISD as compared with the FSD. The cost reduction and improvements in safety associated with the niraparib ISD support its use in clinical practice.

## Summary points

- Based on the results from PRIMA/ENGOT-OV26/GOG-3012 trial (NCT02655016), the niraparib individualized starting dose (ISD) was approved for first-line maintenance therapy in the US. However, the economic impact of the niraparib ISD on the cost of hematologic adverse event (AE) management remains unexplored.
- Grade ≥3 hematologic AEs that occurred in >1% of patients treated with niraparib in the PRIMA primary analysis (thrombocytopenia, anemia, platelet count decreased, neutropenia and neutrophil count decreased) were selected for inclusion in the cost analysis.
- This US-based analysis used the 2017 Agency for Healthcare Research and Quality Healthcare Cost and Utilization Project National Inpatient Sample database to obtain unit costs for each grade ≥3 AE, adjusted to 2020 US dollars.
- AEs were assumed to occur independently of one another, so costs were added to derive the total cost.
- For niraparib, the estimated AE management cost per patient in the US was lower for the ISD than for the fixed starting dose (FSD) for all hematologic AEs.
- The total mean calculated cost per patient was $12,987.71 with the FSD and $6744.93 with the ISD.
- Among the evaluated hematologic AEs, thrombocytopenia had the greatest cost reduction (59.1%).
- Niraparib ISD reduced the total cost of hematologic AE management by 48.1% as compared with the FSD.
- These findings highlight the cost reduction and safety improvements associated with the ISDs of niraparib.
